# Genome-wide comparison of *Corynebacterium diphtheriae* isolates from Australia identifies differences in the Pan-genomes between respiratory and cutaneous strains

**DOI:** 10.1186/s12864-018-5147-2

**Published:** 2018-12-04

**Authors:** Verlaine J. Timms, Trang Nguyen, Taryn Crighton, Marion Yuen, Vitali Sintchenko

**Affiliations:** 1 Centre for Infectious Diseases and Microbiology, Westmead Hospital, PO Box 533, Wentworthville, NSW 2145 Australia; 2 Centre for Infectious Diseases and Microbiology Laboratory Services, ICPMR-Pathology West, Sydney, Australia; 3 Marie Bashir Institute for Infectious Diseases and Biosecurity, Sydney Medical School, The University of Sydney, Sydney, Australia

**Keywords:** Whole genome sequencing, Diphtheria, Vaccine preventable disease, Molecular epidemiology, Pan-genome analysis, Virulence

## Abstract

**Background:**

*Corynebacterium diphtheriae* is the main etiological agent of diphtheria, a global disease causing life-threatening infections, particularly in infants and children. Vaccination with diphtheria toxoid protects against infection with potent toxin producing strains. However a growing number of apparently non-toxigenic but potentially invasive *C. diphtheriae* strains are identified in countries with low prevalence of diphtheria, raising key questions about genomic structures and population dynamics of the species. This study examined genomic diversity among 48 *C. diphtheriae* isolates collected in Australia over a 12-year period using whole genome sequencing. Phylogeny was determined using SNP-based mapping and genome wide analysis.

**Results:**

*C. diphtheriae* sequence type (ST) 32, a non-toxigenic clone with evidence of enhanced virulence that has been also circulating in Europe, appears to be endemic in Australia. Isolates from temporospatially related patients displayed the same ST and similarity in their core genomes. The genome-wide analysis highlighted a role of pilins, adhesion factors and iron utilization in infections caused by non-toxigenic strains.

**Conclusions:**

The genomic diversity of toxigenic and non-toxigenic strains of *C. diphtheriae* in Australia suggests multiple sources of infection and colonisation. Genomic surveillance of co-circulating toxigenic and non-toxigenic *C. diphtheriae* offer new insights into the evolution and virulence of pathogenic clones and can inform targeted public health actions and policy. The genomes presented in this investigation will contribute to the global surveillance of *C. diphtheriae* both for the monitoring of antibiotic resistance genes and virulent strains such as those belonging to ST32.

**Electronic supplementary material:**

The online version of this article (10.1186/s12864-018-5147-2) contains supplementary material, which is available to authorized users.

## Background

Prior to the introduction of toxoid vaccination in 1924, diphtheria was the number one cause of infant death in Australia. Vaccination has meant that infection with *Corynebacterium diphtheriae*, the causative agent of diphtheria, is now a rare occurrence in both Australia and other developed countries around the world [1]. However *C. diphtheriae* can also exist in a toxin negative form which is not covered by immunisation. Toxin-negative isolates of *C. diphtheriae* have been revealed to be associated with prosthetic and native valve endocarditis and significantly and more importantly have been increasingly detected in clinical samples [2, 3]. This increase in detection of this bacterium is thought to be due to the uptake of matrix-assisted laser desorption/ionisation time-of-flight mass spectrometry (MALDI-TOF MS) identification. The growing numbers of emerging non-toxigenic but potentially invasive *C. diphtheriae* isolates identified by diagnostic and public health laboratories in countries with low prevalence of diphtheria raises concerns about other virulence factors and the population dynamics of the species. Since the publication of the first complete genome sequence of *C. diphtheriae* [4] the phylogeographical structure of this species and the role of iron-uptake systems, adhesions and fimbrial proteins in virulence have become key questions that need addressing [5]. Furthermore, potent toxigenic variants may emerge from local strains being lysogenized by a toxin gene carrying corynebacteriophage [6].

The most virulent *C. diphtheriae* are those that possess the toxin gene and produce diphtheria toxin (DT), one of the most potent exotoxins known. In order for *C. diphtheriae* to produce DT it must be lysogenized by a corynebacteriophage carrying a toxin gene (*tox*). The two most common bacteriophages known to infect *C. diphtheriae* are corynephage β and ω. However corynebacteriophages from *C. ulcerans* can also carry *tox* gene homologs and it remains unknown whether bacteriophages of *C. ulcerans* can lysogenize *C. diphtheriae* [7]. In addition, very little is known about whether other toxin gene variants exist and the role that other *C. diphtheriae* pathogenic factors may play. Toxin production is regulated by a repressor on the bacterial chromosome, DtxR in response to the amount of free iron available in the local environment [8]. DtxR is also responsible for the regulation of a wide range of other genes used for colonisation, nutrient acquisition and persistence and has been shown to vary among strains [9]. Factors involved in iron metabolism and virulence determinants such as pili may also vary among strains and contribute to the success of certain clones [10]. Adherence properties are now seen to be major virulence properties for *C. diphtheriae* and can vary widely between strains. Adherence is largely governed by the presence of complete Spa pilus gene clusters, some of which are carried on variable pathogenicity islands [11].

The advancement of whole genome sequencing (WGS) has led to revolutionary change for public health laboratory surveillance, opening up the potential to describe outbreaks in high resolution and to explore potential transmission routes [7, 8]. WGS can be used to describe strain differences in terms of virulence properties such as DT, Spa pilus gene clusters and to investigate genes that may be associated with attributes such as disease site or an administrative local health district (LHD), for example. The aim of this study was to examine genomic variation among *C. diphtheriae* isolates identified in the most populous state of Australia and referred to our laboratory for diphtheria toxin testing over a 12 year period. We examined whether *C. diphtheriae* transmission has been occurring locally and whether recent strains contained toxin homologs undetectable with existing assays. We also investigated if other pathogenic factors such as pili variation were contributing to possible local transmission.

## Methods

### Bacterial isolates and molecular subtyping

All *C. diphtheriae* clinical isolates collected between January 2004 and January 2016 by the Microbial Identification Laboratory NSW Health Pathology at the Centre for Infectious Diseases and Microbiology, Westmead Hospital were included in the study. Personal data were removed from all material related to the study to protect the anonymity of the patients. The only data available at sample collection was age, gender, disease site and region, with region defined as local health district (LHD; there were four, LHD-1 to LHD-4). The LHD was where the isolate of *C. diphtheriae* was established and referred from. Clinical management was not changed. This study did not involve identifiable human material or identifiable patient data and therefore was not governed by the Declaration of Helsinki and ethical approval was not deemed necessary. Bacterial isolates were cultured on horse blood agar and incubated aerobically at 37 °C. Bacterial isolates were identified as *C. diphtheriae* using MALDI-TOF on a Bruker microflex LT (Bruker Daltonik GmbH) with a cut-off of 2.2 with details of this procedure published previously [12]. The biotype was determined biochemically using the API® Coryne Strip (API bioMérieux). Toxin studies were carried out using the modified Elek test [13] and PCR for the diphtheria toxin gene [14].

### DNA extraction and whole genome sequencing (WGS)

Genomic DNA was extracted from pure cultures using the DNeasy Blood & Tissue Kit (QIAGEN). Type strains *C. diphtheriae* ATCC 27010 (C7(−)) and ATCC 13812 (PW8) were included for comparison of assembly and typing pipelines. Paired-end indexed libraries of 150 bp in length were prepared from an input of 1 ng of purified DNA with the Nextera XT kit (Illumina) as per manufacturer’s instructions. DNA libraries were then sequenced using the NextSeq 500 Instrument (Illumina).

### Genome assembly and analysis

The quality of the sequence data was assessed using FastQC (https://www.bioinformatics.babraham.ac.uk/projects/fastqc/). Sequencing reads were assembled with Spades [15] and annotated with Prokka [16]. Multiple locus sequence typing (MLST) was performed with seven loci by uploading assembled fasta sequences to the PubMLST *Corynebacterium diphtheriae* database (http://pubmlst.org/cdiphtheriae/). In addition, pan-genome assessment and visualisation was performed using default parameters in Roary [17] including alignment using Multiple Alignment using Fast Fourier Transform (MAFFT) [18] and tree building with FastTree [19]. To look for associations between pan-genome gene content and available metadata, we used Scoary version 1.4.0 [20] with default parameters. Genes with corrected *p*-value (Benjamini-Hochberg) of association below 0.05 were considered significant. For the analysis, we used disease site (cutaneous, respiratory or blood), age, gender and local health district (LHD 1–4) as the traits of interest. Results were visualised by taking the outputs from Roary (phylogenetic tree and genes presence absence files) and uploading to the Phandango website [21]. Further visualisation of phylogeny with metadata (site, LHD, ST) was done with Interactive Tree of Life (iTOL) [22].

To identify Single nucleotide polymorphisms (SNPs), files were imported into Geneious (8.0.4) and mapped to the reference *C. diphtheriae* NCTC 13129 using the bwa plugin (version 0.7.10). Quality based variant detection was performed using CLC Genomics Workbench v.7.0 (CLC bio Aarhus, Denmark). Variant detection thresholds were set for a minimum coverage of 10 and minimum variant frequency of 75%. SNPs were excluded if they were in regions with a minimum fold coverage of < 10, within 10-bp of another SNP or < 15-bp from the end of a contig. Maximum likelihood phylogenetic trees were constructed from SNP matrices using the GTR model with 100 bootstrap replications. Antibiotic resistance was predicted using Abricate (version 0.5) with settings to use all seven resistance prediction databases [23]. BLAST comparisons to search for toxin homologs was performed with the following toxin homologs: Corynephage beta A and B subunit (NCBI accession number P00588), Corynephage omega Diphtheria toxin (accession number P00587), Corynephage beta Diphtheria toxin homolog (accession number P00589) and *C. ulcerans* Diphtheria toxin homologs (accession number Q6YIX9 and Q5IL09). The homology of *dtx*R and all the genes from the Spa pilus gene clusters (A, D &H) (as defined previously [11, 24, 25]) was also determined using the BLAST suite with orthologs defined as those that had at least 50% length and 75% homology compared to corresponding gene in NCTC13129. Siderophore clusters were compared using antiSMASH [26]. The genomic data have been deposited in the NCBI Sequence Read Archive (SRA) (http://www.ncbi.nlm.nih.gov/Traces/sra/) under accession number (SRP134141).

## Results

**Fig. 1:**
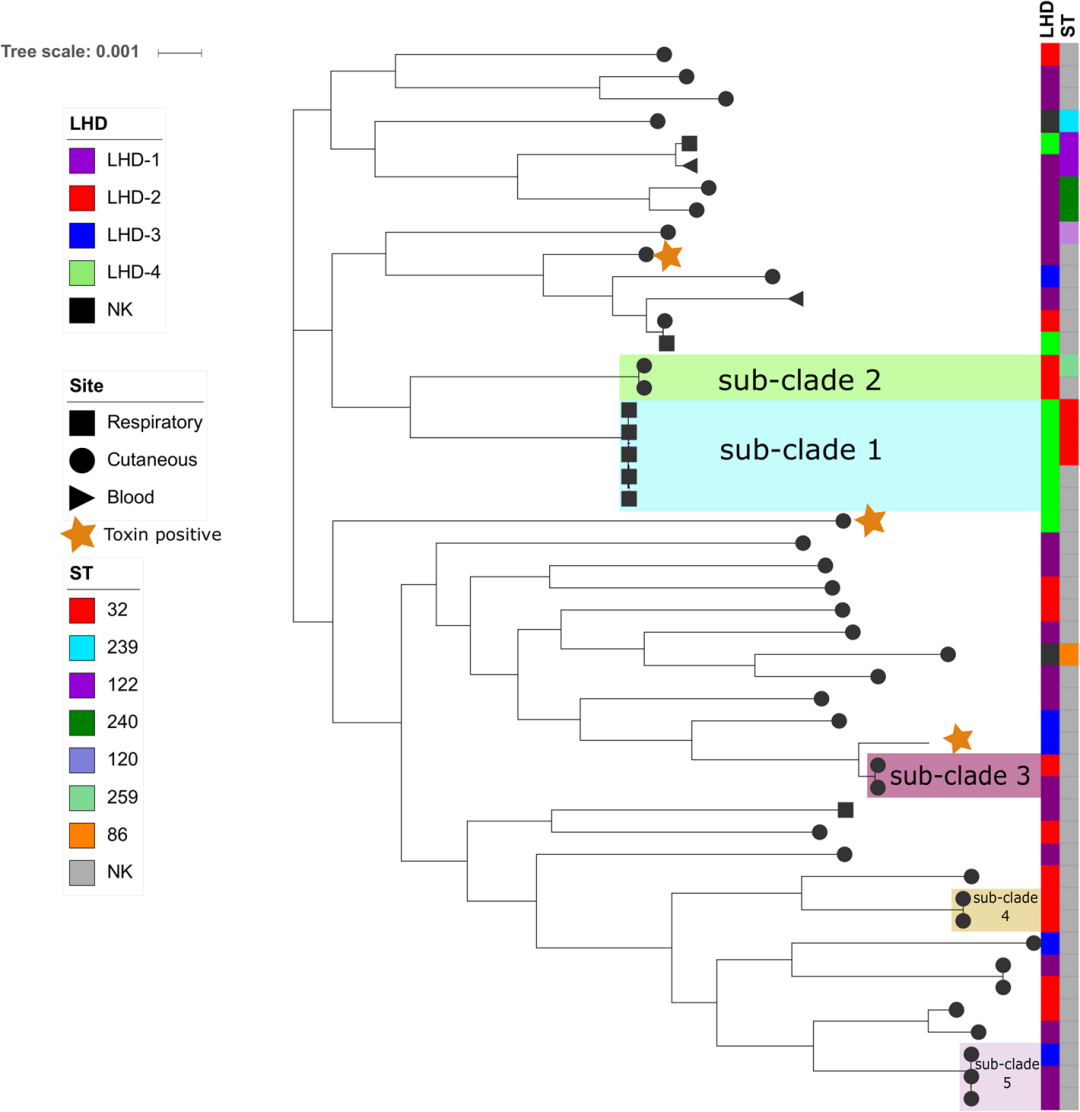
Core phylogenetic tree with Local Health District (LHD), sequence type (ST), disease site and toxin gene presence marked. Coloured shaded blocks highlight sub-clades identified by ST and date isolated. Image prepared with iTOL [22]

Forty eight isolates were recovered from symptomatic patients between 2004 and 2016 with 36 of these collected in 2014–2016. Three isolates were toxigenic (all biovar. *mitis*) and identified in 2015 (Table 1). Relative to the reference genome, NCTC13129, 170,262 SNPs were detected across all isolates. The genome size was in the range of 2.2–2.7 Mb with an average G + C ratio of 53%. MLST typing revealed that some isolates shared the same or had similar sequence type (ST) (Fig. 1, Additional file 1). Core genome analysis on de novo assembled genomes identified 1384 core genes, 354 ‘soft core’ genes (present between 95 and 99% of strains), 888 ‘shell genes’ (present in 15–95% of strains), 5177 cloud genes (present between 0 and 15% of strains) and a total pan-genome of 7803 genes. Only those strains that were known to be toxin positive (CD33, CD29 and CD38) demonstrated the toxin gene or any homologs by BLAST (Fig. 1). No variability was observed in the *dtxR* gene for any strain in this study. Three unrelated isolates, CD1, CD40 and CD42 contained the erythromycin resistance gene *ermX*.

**Table 1:** *C. diphtheriae* strains analysed in this study

Isolate	Biotype	Year isolated	Gender	Age (years)	Origin	LHD	Sequence Type
CD2	*gravis*	2007	NK	NK	Cutaneous (foot)	NK	239
CD3	*gravis*	2007	M	53	Blood	4	122
CD4	*mitis*	2008	NK	NK	Cutaneous (leg)	NK	86
CD1	*gravis*	2009	F	19	Respiratory	4	New^b^
CD5	*gravis*	2012	F	13	Blood	1	122
CD6	*mitis*	2012	M	20	Cutaneous (leg)	1	New^b^
CD7	*mitis*	2012	M	63	Cutaneous (site unknown)	2	New^b^
CD8	*gravis*	2012	M	20	Respiratory	4	32
CD9	*mitis*	2012	F	93	Cutaneous (site unknown)	1	New^b^
CD12	*gravis*	2013	F	16	Respiratory	4	32
CD10	*gravis*	2013	M	65	Cutaneous (leg)	1	240
CD11	*mitis*	2013	M	61	Cutaneous (site unknown)	2	New^b^
CD15	*gravis*	2014	F	23	Blood	1	New^b^
CD24	*mitis*	2014	M	72	Cutaneous (leg)	1	New^b^
CD13	*mitis*	2014	M	66	Cutaneous (foot)	1	New^b^
CD14	*mitis*	2014	M	25	Cutaneous (foot)	2	259
CD20	*mitis*	2014	M	19	Cutaneous (hand)	2	New^b^
CD17	*mitis*	2014	M	88	Cutaneous (site unknown)	3	New^b^
CD16	*mitis*	2014	M	46	Cutaneous (site unknown)	1	New^b^
CD18	*mitis*	2014	M	41	Cutaneous (site unknown)	1	New^b^
CD19	*gravis*	2014	F	27	Respiratory	4	32
CD23	*gravis*	2014	F	25	Respiratory	4	New^b^
CD26	*gravis*	2014	F	25	Respiratory	4	New^b^
CD27	*gravis*	2014	M	25	Cutaneous (arm)	2	New^b^
CD21	*mitis*	2014	M	19	Cutaneous (leg)	2	New^b^
CD22	*mitis*	2014	F	43	Cutaneous (site unknown)	2	New^b^
CD25	*mitis*	2014	M	70	Cutaneous (leg)	3	20
CD28	*gravis*	2014	M	38	Cutaneous (site unknown)	1	147
CD32	*mitis*	2015	M	34	Cutaneous (site unknown)	1	New^b^
CD29^a^	*gravis*	2015	M	18	Cutaneous (foot)	1	120
CD33^a^	*gravis*	2015	M	89	Cutaneous (site unknown)	4	59
CD34	*gravis*	2015	F	44	Cutaneous (site unknown)	2	New^b^
CD31	*mitis*	2015	F	45	Cutaneous (site unknown)	3	New^b^
CD30	*mitis*	2015	M	6	Cutaneous (site unknown)	1	New^b^
CD35	*gravis*	2015	M	41	Cutaneous (leg)	1	New^b^
CD37	*mitis*	2015	M	46	Cutaneous (ankle)	3	New^b^
CD38^a^	*gravis*	2015	M	46	Cutaneous (ankle)	3	381
CD36	*mitis*	2015	F	20	Cutaneous (site unknown)	2	New^b^
CD39	*mitis*	2015	M	59	Cutaneous (site unknown)	1	New^b^
CD40	*mitis*	2015	M	35	Cutaneous (penile ulcer, underlying syphilis)	2	6
CD41	*mitis*	2015	M	67	Cutaneous (site unknown)	2	New^b^
CD42	*mitis*	2015	M	27	Cutaneous (site unknown)	1	5
CD44	*gravis*	2016	M	43	Cutaneous (wound unknown site)	1	240
CD52	*mitis*	2016	M	61	Cutaneous	1	New^b^
CD45	*mitis*	2016	M	18	Cutaneous (foot)	2	New^b^
CD46	*mitis*	2016	M	46	Cutaneous	2	New^b^
CD47	*mitis*	2016	M	14	Cutaneous (penis, circumcision wound)	1	New^b^
CD43	*mitis*	2016	M	55	Cutaneous (site unknown)	1	New^b^
ATCC13812	*gravis*	1896	NK	NK	Respiratory	–	44
ATCC27010	*mitis*	1954	NK	NK	Respiratory	–	26

^a^denotes toxin positive strains; *NK* Not Known

^b^all new sequence types are unique (see Additional file 1)

We tested for relative differences in pan-genome content between isolates and available metadata using Scoary, which performs a genome-wide association study (GWAS) using gene presence and absence. After correcting for multiple-hypothesis testing using the Benjamini-Hochberg procedure we did not identify any genes that were significantly associated with age and gender. However associations were found for LHD 4 (55 genes), respiratory infection (122 genes) and cutaneous infection (102 genes) Additional file 2. All but two of the genes associated with LHD-4 were also found to be significantly associated with respiratory and cutaneous infection (Additional file 2). In the pan-genome analysis, 22 genes from gene group I were found to have a significant association with respiratory infection and were largely made up of hypothetical proteins but did contain bacteriophage proteins (Fig. 3, Additional files 2 and 3).

In order to identify strains that were possibly related, sub-clades were defined as having the same or similar (up to one allele difference) MLST type and isolation in the same or consecutive year. Based on these criteria, six sub-clades were identified and the features of these sub-clades are outline below.

**Fig. 2:**
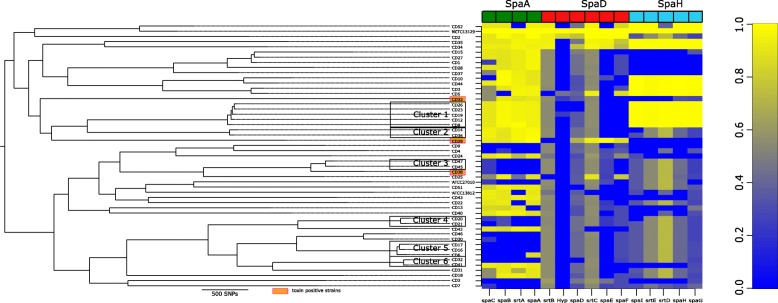
Maximum likelihood tree based on genome-wide SNP detection of reads mapped to reference NCTC13129. Branch lengths correspond to numbers of nucleotide substitutions per site. The heatmap shows Spa pilus gene clusters when compared to the reference NCTC11329 with high homology shown in yellow, absence or poor homology shown in blue

**Fig. 3:**
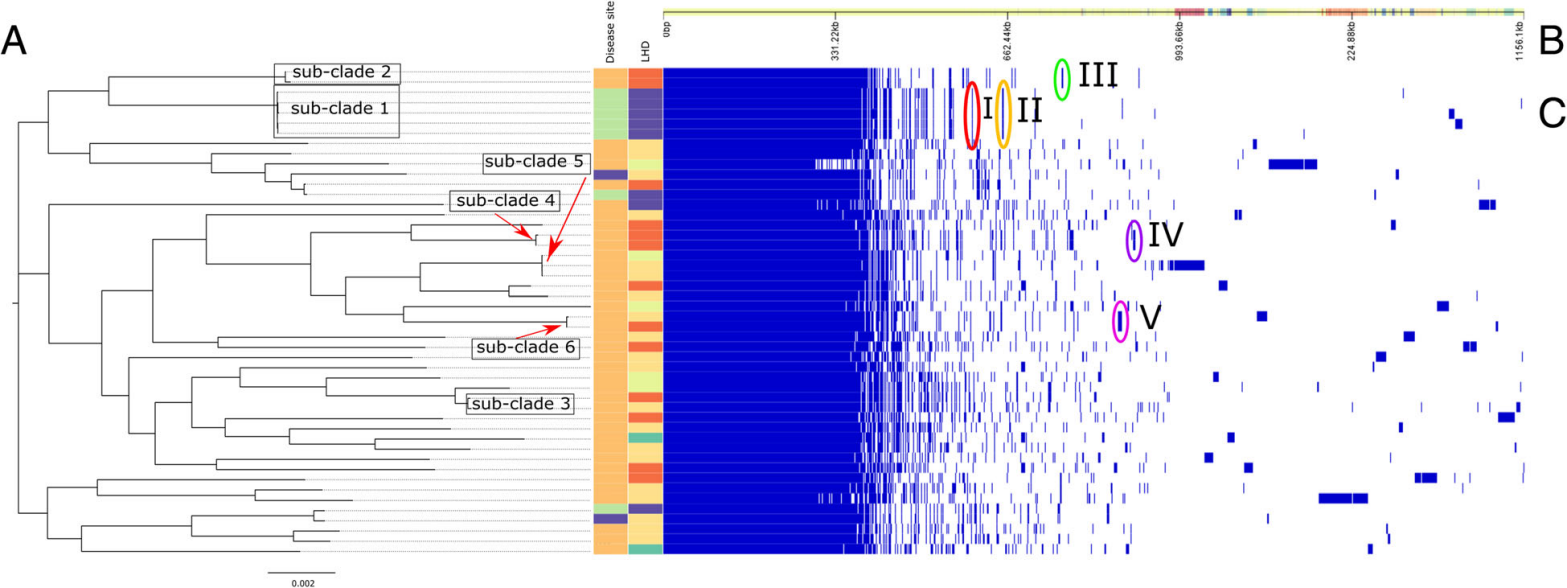
The gene groups unique to sub-clades according to pan-genome analysis with core genome phylogenetic tree (**a**). The top panel (**b**) shows a single representative nucleotide sequence inferred for each gene of the pangenome. The middle panel (**c**) displays presence (blue) or absence (white) of blocks relative to genes and contigs in the pan-genome and metadata on disease site and health region (LHD). Disease site is classified as respiratory (green), cutaneous (orange) and blood (purple). There were four LHD regions identified as LHD-1 (orange), LHD-2 (red), LHD-3 (yellow) and LHD-4 (purple). Unique gene groups found in defined sub-clades have been circled and numbered accordingly; gene group I (red) - transposable elements and other proteins found in sub-clade 1; gene group II (orange) - 11 genes containing transposons, unique outer membrane proteins and a phenazine biosynthesis protein (PhzF); gene group III (green) – genes unique to sub-clade 2 containing *tetO*; gene group IV - *sul1*, genes for a fimbrial subunit type 1, sulphur carrying protein (ThiS), inner membrane transporter protein (RhIA) and VRR-NUC domain; gene group IV (purple) – genes unique to sub-clade 6 containing *sdpA* and *sdpB*, an integrase, von Willebrand factor type A domain protein and a putative transposon Tn552, all shown to be part of a NRPS/ PKS module and another NRPS module containing homologs of *mbtB*, *irtA* and *irtB*. The image was prepared using Phandango [21]

Within four sub-clades (sub-clades 1, 2, 4 and 5) some isolates were from patients that appeared to be geographically linked (Fig. 1 and Fig. 2). Sub-clade 1 contained strains CD12, CD19, CD8 and CD23/CD26 (with CD23/CD26 isolated from the same patient, retrieved from samples taken 7 weeks apart). This sub-clade had the in silico MLST profile for ST32; *atpA*-3, *dnaE*-1, *dnaK*-18, *fusA*-4, *leuA*-13, *odhA*-3, *rpoB*-5, with the exception of strains CD23/CD26 that differed by one nucleotide (C to T) in the *dnaK* locus (Table 1, Fig. 1 and Additional file 1). All strains of this sub-clade were from respiratory samples of adult patients residing in the same LHD (Figs. 1 and 3).

Analysis of the pan-genome showed that sub-clade 1 contained unique genes denoted by gene groups I and II (Fig. 3). The first (gene group I) were mainly hypothetical proteins; however, two were annotated as transposable elements, one ATPase and a modification methylase that was not found in any other isolates in this study (Fig. 3). An additional 11 genes (gene group II) were also unique and mostly hypothetical but again contained transposons, unique putative outer membrane proteins and a phenazine biosynthesis protein (PhzF family) (Additional file 3). Analysis of SpaA, SpaD and SpaH pilus gene clusters showed that all were present in this sub-clade, even though the SpaD gene cluster had low homology to the reference strain (Fig. 2).

Sub-clade 2 consisted of two isolates that differed by only one MLST allele. The first isolate CD14 was ST259 (*atpA*-3, *dnaE*-1, *dnaK*-12, *fusA*-1, *leuA*-42, *odhA*-16, *rpoB*-31), while isolate CD36 differed by one nucleotide in the *dnaK* locus. Both isolates were predicted to be resistant to phenicol, sulphonamide and tetracycline. Pan-genome analysis showed that the two isolates from this sub-clade contained the *tetO* gene predicting resistance to tetracycline (III Fig. 3). This sub-clade did not contain the *spaE* or *spaF* gene and had variable homology in SpaH (Fig. 2).

Sub-clade 3 consisted of isolates CD47 and CD45 and both isolates were from teenage males (Fig. 1). These isolates had the MLST profile of *dnaK*-8, *fusA*-53, *leuA*-3, *odhA*-5, *rpoB*-13 which closely resembled ST381. No geographic link was determined and no antibiotic resistance genes were identified. Pan-genome analysis did not show any unique genes common to both strains. Like sub-clade 2, strains from this sub-clade did not contain the *spaF* gene and had variable homology in both SpaD and SpaH (Fig. 2).

Sub-clade 4 was represented by isolates CD20 and CD21 from two patients (both the same age) residing in the same LHD (Fig. 1). The two isolates represented a new ST (*atpA*-13, *dnaE*-2, *dnaK*-32, *fusA*-33, *leuA*-no match, *odhA*-1, *rpoB*-21). These strains had a unique gene group (IV) that included a phage that contained the sulphonamide resistance gene *sul1*, as well as genes for a fimbrial subunit type 1, sulphur carrying protein (ThiS), inner membrane transporter protein (RhIA) and VRR-NUC domain protein to name a few (Fig. 3, Additional file 3). All three Spa pilus gene clusters were highly variable in this sub-clade and did not have significant homology with the reference NCTC 13129 (Fig. 2).

In the fifth sub-clade, isolates CD17, CD16 and CD6 represented a new ST with identical loci. All isolates were from males (age range 20–88 years) from the same LHD (Fig. 1). No markers of antibiotic resistance were detected and no unique genes were identified in this sub-clade. Similar to sub-clade 4, all three Spa pilus gene clusters were highly variable in this sub-clade and did not have significant homology with the reference NCTC 13129 (Fig. 2).

Sub-clade 6 consisted of isolates CD32 and CD41, with identical MLST alleles. No antibiotic resistance genes were predicted. Pangenomic analysis demonstrated unique genes for these two strains that contained *sdpA* and *sdpB*, both sporulation-delaying proteins, an integrase, von Willebrand factor type A domain protein and a putative transposon Tn552, all of which were shown to be part of a non-ribosomal peptide (NRPS)/ polyketide synthase (PKS) module unique to these strains (Fig. 3, gene group V). In addition, these strains contained a large NRPS module with homologs to *mbtB*, *irtA* and *irtB*, genes known for iron regulation and survival in *M. tuberculosis* [27].

**Fig. 4:**
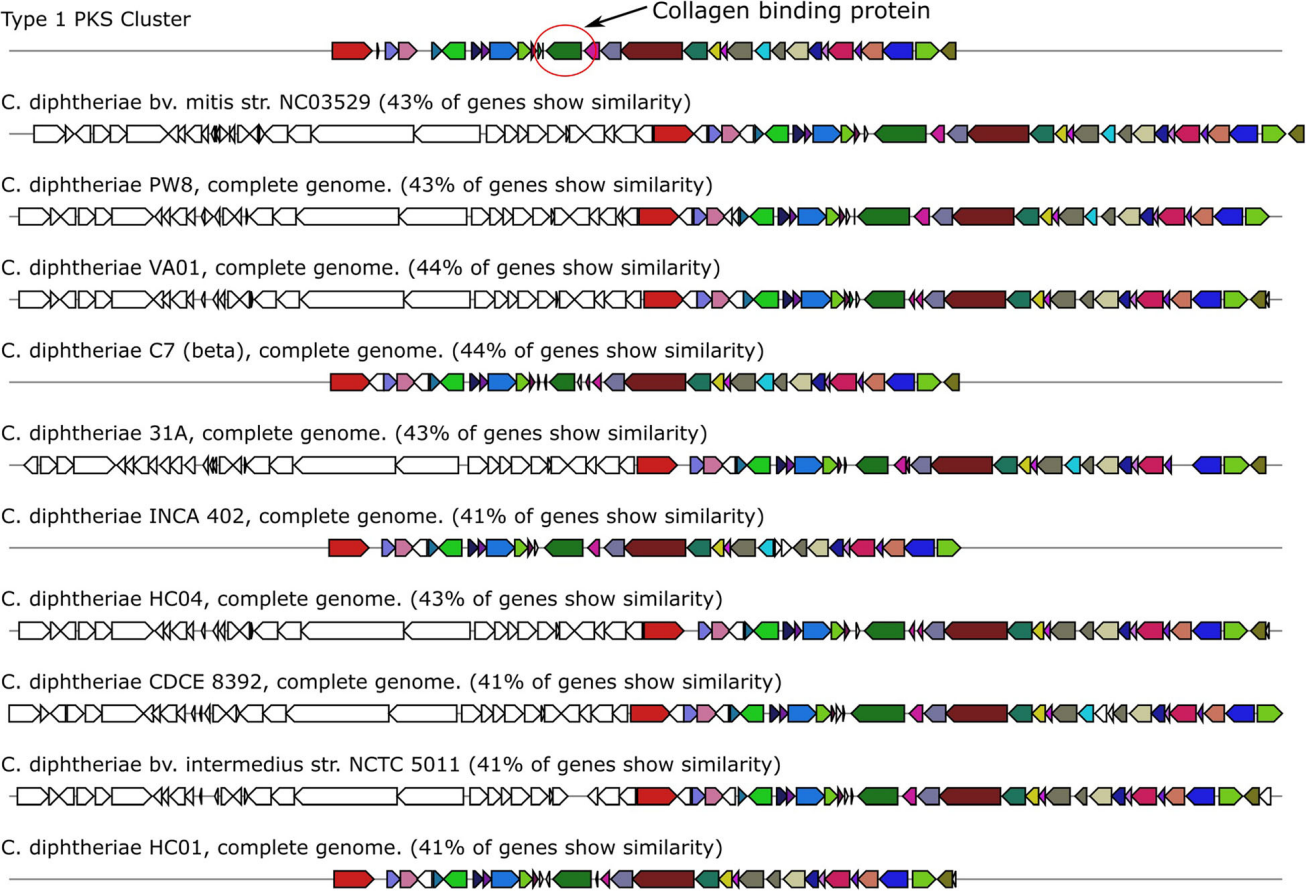
The type 1 PKS cluster of *C. diphtheriae*. The collagen binding protein is indicated by the red circle and corresponds to the same green gene in each cluster. The homology of the cluster is indicated for a selection of well characterised *C. diphtheriae* genomes on Genbank, with the closest homology across the cluster. Siderophore modules were compared and image generated using antiSMASH [26]

Interestingly, NRPS/PKS modules contained small variations among strains; however most notable was an additional gene present in the Type 1 PKS gene cluster that was present in strains associated with systemic and cutaneous infections and was absent in all respiratory isolates in our sample. This protein was annotated as a putative collagen binding protein and showed high homology (89–94%) to similar proteins in *C. diphtheriae* isolates from cutaneous infections but low homology (< 84%) in respiratory strains (Fig. 4).

Genome-wide comparison of isolates also uncovered concurrent infection in one patient with two genomically distinct strains of *C. diphtheriae* recovered from the same wound (Table 1). The first isolate CD37 was *tox* negative and biotype *gravis* while the second isolate CD38 was toxigenic and biotype *mitis* (Table 1). WGS analysis was initially performed on DNA from the first isolate and the toxin gene could not be found despite the PCR assay detecting the diphtheria toxin gene. The stored isolate was then resuscitated and 10 colonies were selected. PCR for the toxin gene and WGS was performed on all 10 selected isolates, including a sweep from the original plate and all of them contained the toxin gene and all had the same genome, CD38 with none matching the original genome, CD37. Isolates CD37 and CD38 were not related to each other according to the analysis employed in this study (Figs. 1 and 2). Further, isolate CD37 did not group with any other genome in this dataset indicating that it was not a contaminant from other patients.

## Discussion

This report describes the epidemiology of toxigenic and non-toxigenic *C. diphtheriae* in New South Wales (NSW) over a 12 year period. Apart from several sub-clades described above, most strains represented very diverse STs reflecting multiple sources of infection and possibly overseas-acquired cases. There is little data on the evolution of *C. diphtheriae*, particularly on current *tox*^*−*^ strains. While the number of notifiable cases has remained low during this period, the number of isolates referred to our laboratory for diphtheria toxin testing has risen remarkably with 36 of the 47 isolates collected in 2014–2016. This has also been reported by other developed countries and like this study, most isolates are found to be non-toxigenic [28].

This study adds important insights into the evolution of *C. diphtheriae*, particularly on current *tox*^*−*^ strains in countries with high uptake of diphtheria toxoid vaccines. As immunisation is achieved using vaccine containing diphtheria toxoid, it does not confer immunity to non-toxigenic strains. Infection with non-toxigenic *C. diphtheriae* can still result in respiratory, cutaneous or invasive infections [1, 29] with the cutaneous route speculated to be the more efficient in transmission [30]. Our pan-genome analysis showed that some genes had a significant association to either cutaneous or respiratory infection. However a proportion of these genes were associated with both respiratory and cutaneous infections indicating that the presence of these genes are most likely associated with highly virulent strains. Nevertheless, 22 genes were found to be uniquely associated with respiratory infections. These genes were largely annotated as hypothetical proteins but were found to contain phage proteins and were present in strains typed as ST32, a ST suspected to have enhanced virulence.

The investigation of population structure using traditional MLST profiling, SNP-based mapping and pan-genome analysis has identified apparent groups of cases of infection and indicated surprisingly, local acquisition of *C. diphtheriae*. For example, ST32, was found in four patients, all from the same LHD and had a similar age range (16–27 years). ST32 has been reported previously and the success of this clone is thought to be due to its superior adherence properties [24]. The adhesion rate for ST32 is reported to be 7.34 + 2.33%, compared to other *tox*^+^*C. diphtheriae* strains that have an adhesion rate of 0.34 + 0.05% [31]. We therefore examined the pilus gene clusters of our ST32 isolates and reconfirmed that the ST32 strains contained the SpaA, SpaD and SpaH pilus gene clusters although the ST32 isolated in our study had poor homology to SpaD from NCTC13139. Pili are essential for the establishment of infection, particularly in the respiratory tract, and are contained on a pathogenicity island in *C. diphtheriae* [31]. They are also known to vary between strains and possibly contribute to the success of certain clones. SpaA type pili are involved in adhesion to pharyngeal epithelial cells while SpaD and SpaH interact with laryngeal and lung epithelium and are highly heterogeneous across strains [5]. Loss of *srtA* and/or genes from *spaB* or *spaC* (all from the SpaA pilus gene cluster) equates to loss of adhesion [32]. Interestingly, Sub-clade 1 was the only sub-clade that contained all three pilus gene clusters and all strains were recovered from patients with respiratory disease.

Antibiotic resistance in *C. diphtheriae* remains relatively uncommon; however, a recent report found that *C. diphtheriae* isolates showed a decreased susceptibility to penicillin and resistance to tetracycline in Rio de Janeiro [33]. Multidrug resistant isolates involved in cutaneous and respiratory diphtheria have also been described [34, 35]. Further studies have reported that penicillin resistance has contributed to treatment failure [36]. Our findings suggest that antibiotic resistance is uncommon among *C. diphtheriae* in Australia. Isolates from sub-clade 2 appeared to carry the *tetO* gene, encoding a protein which protects the ribosome from the translation inhibition action of tetracyclines. Erythromycin resistance was predicted in three strains (none of which belonged to a sub-clade) by the presence of *ermX*.

The emerging role of siderophores as important virulence factors of *C. diphtheriae*, deserves special attention. Iron acquisition mechanisms in pathogenic bacteria are known to contribute to survival under “nutritional immunity” a mechanism induced by the host to reduce pathogen cell replication and growth [37]. Siderophores are encoded by large non-ribosomal peptide modules known as NRPS/PKS modules which confer survival ability in nutrient variable conditions, particularly in establishing and maintaining infection. We demonstrated variability in the siderophore modules between the STs. Interestingly, strains from cutaneous and systemic infections contained an additional gene encoding a collagen binding protein that had high homology among strains isolated from cutaneous or blood infections but low homology (< 84%) among strains isolated from respiratory infections. The contribution of this collagen binding protein (in combination with an increase in adherence mechanisms) to the success of particular toxin-negative strains warrants further study.

## Conclusions

The genomic diversity of toxigenic and non-toxigenic strains of *C. diphtheriae* in Australia suggests multiple sources of human infection and colonisation. Core and accessory genomes of *C. diphtheriae* strains colonising different ecological niches have significant differences and have virulence mechanisms that modulate their fitness as pathogens. Given the growing numbers of *C. diphtheriae* isolates being identified in diagnostic laboratories it has become important to closely monitor non-toxigenic strains of *C. diphtheriae.* The findings of additional phage or virulence factors conferring potential advantage on strains of *C. diphtheriae* can be of public health concern as a vaccinated population would have no immunity to new strains as vaccines contain the diphtheria toxoid only.

## Additional files

Additional file 1: The MLST allele designations and assembly statistics for *C. diphtheriae* genomes. (XLSX 17 kb)

Additional file 2: Genes associated with traits, LHD, respiratory infection and cutaneous infection. (XLSX 59 kb)

Additional file 3: Listed genes from pan-genome analysis. (XLSX 13 kb)

## References

[1] Adler NR, Mahony A, Friedman ND (2013). Diphtheria: forgotten, but not gone. Intern Med J.

[2] Belko J, Wessel DL, Malley R (2000). Endocarditis caused by *Corynebacterium diphtheriae:* case report and review of the literature. Pediatr Infect Dis J.

[3] Doyle CJ, Mazins A, Graham RMA, Fang N-X, Smith HV, Jennison AV (2017). Sequence analysis of toxin gene–bearing *Corynebacterium diphtheriae* strains, Australia. Emerg Infect Dis.

[4] Cerdeno-Tarraga AM, Efstratiou A, Dover LG, Holden MT, Pallen M, Bentley SD, Besra GS, Churcher C, James KD, De Zoysa A (2003). The complete genome sequence and analysis of *Corynebacterium diphtheriae* NCTC13129. Nucleic Acids Res.

[5] Broadway MM, Rogers EA, Chang C, Huang IH, Dwivedi P, Yildirim S, Schmitt MP, Das A, Ton-That H (2013). Pilus gene pool variation and the virulence of *Corynebacterium diphtheriae* clinical isolates during infection of a nematode. J Bacteriol.

[6] Mokrousov I (2009). *Corynebacterium diphtheriae*: genome diversity, population structure and genotyping perspectives. Infect Genet Evol.

[7] Meinel DM, Margos G, Konrad R, Krebs S, Blum H, Sing A (2014). Next generation sequencing analysis of nine *Corynebacterium ulcerans* isolates reveals zoonotic transmission and a novel putative diphtheria toxin-encoding pathogenicity island. Genome Med.

[8] Meinel DM, Kuehl R, Zbinden R, Boskova V, Garzoni C, Fadini D, Dolina M, Blumel B, Weibel T, Tschudin-Sutter S (2016). Outbreak investigation for toxigenic *Corynebacterium diphtheriae* wound infections in refugees from Northeast Africa and Syria in Switzerland and Germany by whole genome sequencing. Clin Microbiol Infect.

[9] Trost E, Ott L, Schneider J, Schroder J, Jaenicke S, Goesmann A, Husemann P, Stoye J, Dorella FA, Rocha FS (2010). The complete genome sequence of *Corynebacterium pseudotuberculosis* FRC41 isolated from a 12-year-old girl with necrotizing lymphadenitis reveals insights into gene-regulatory networks contributing to virulence. BMC Genomics.

[10] Puliti M, von Hunolstein C, Marangi M, Bistoni F, Tissi L (2006). Experimental model of infection with non-toxigenic strains of *Corynebacterium diphtheriae* and development of septic arthritis. J Med Microbiol.

[11] Iwaki M, Komiya T, Yamamoto A, Ishiwa A, Nagata N, Arakawa Y, Takahashi M (2010). Genome organization and pathogenicity of *Corynebacterium diphtheriae* C7(−) and PW8 strains. Infect Immun.

[12] Vila J, Juiz P, Salas C, Almela M, de la Fuente CG, Zboromyrska Y, Navas J, Bosch J, Aguero J, de la Bellacasa JP (2012). Identification of clinically relevant *Corynebacterium* spp., *Arcanobacterium haemolyticum*, and *Rhodococcus equi* by matrix-assisted laser desorption ionization-time of flight mass spectrometry. J Clin Microbiol.

[13] Engler KH, Glushkevich T, Mazurova IK, George RC, Efstratiou A (1997). A modified Elek test for detection of toxigenic corynebacteria in the diagnostic laboratory. J Clin Microbiol.

[14] Moore C, Ratcliff RM, Lanser JA (1994). Determination of toxigenicity of *Corynebacterium diphtheriae* by PCR. ASM Australia: 1994.

[15] Bankevich A, Nurk S, Antipov D, Gurevich AA, Dvorkin M, Kulikov AS, Lesin VM, Nikolenko SI, Pham S, Prjibelski AD (2012). SPAdes: a new genome assembly algorithm and its applications to single-cell sequencing. J Comput Biol.

[16] Seemann T (2014). Prokka: rapid prokaryotic genome annotation. Bioinformatics.

[17] Page AJ, Cummins CA, Hunt M, Wong VK, Reuter S, Holden MT, Fookes M, Falush D, Keane JA, Parkhill J (2015). Roary: rapid large-scale prokaryote pan genome analysis. Bioinformatics.

[18] Katoh K, Asimenos G, Toh H (2009). Multiple alignment of DNA sequences with MAFFT. Methods Mol Biol.

[19] Price MN, Dehal PS, Arkin AP (2010). FastTree 2--approximately maximum-likelihood trees for large alignments. PLoS One.

[20] Brynildsrud O, Bohlin J, Scheffer L, Eldholm V (2016). Rapid scoring of genes in microbial pan-genome-wide association studies with Scoary. Genome Biol.

[21] Hadfield J, Croucher NJ, Goater RJ, Abudahab K, Aanensen DM, Harris SR. Phandango: an interactive viewer for bacterial population genomics. Bioinformatics. 2017;34(2):292–3. 10.1093/bioinformatics/btx610 PMC5860215 29028899

[22] Letunic I, Bork P (2016). Interactive tree of life (iTOL) v3: an online tool for the display and annotation of phylogenetic and other trees. Nucleic Acids Res.

[23] Abricate. https://github.com/tseemann/abricate. Accessed Feb 2018.

[24] Sangal V, Blom J, Sutcliffe IC, von Hunolstein C, Burkovski A, Hoskisson PA (2015). Adherence and invasive properties of *Corynebacterium diphtheriae* strains correlates with the predicted membrane-associated and secreted proteome. BMC Genomics.

[25] Trost E, Blom J, Soares Sde C, Huang IH, Al-Dilaimi A, Schroder J, Jaenicke S, Dorella FA, Rocha FS, Miyoshi A (2012). Pangenomic study of *Corynebacterium diphtheriae* that provides insights into the genomic diversity of pathogenic isolates from cases of classical diphtheria, endocarditis, and pneumonia. J Bacteriol.

[26] Weber T, Blin K, Duddela S, Krug D, Kim HU, Bruccoleri R, Lee SY, Fischbach MA, Muller R, Wohlleben W (2015). antiSMASH 3.0-a comprehensive resource for the genome mining of biosynthetic gene clusters. Nucleic Acids Res.

[27] Ratledge C, Dover LG (2000). Iron metabolism in pathogenic bacteria. Annu Rev Microbiol.

[28] Wren MW, Shetty N (2005). Infections with *Corynebacterium diphtheriae*: six years’ experience at an inner London teaching hospital. Br J Biomed Sci.

[29] Efstratiou A, Tiley SM, Sangrador A, Greenacre E, Cookson BD, Chen SC, Mallon R, Gilbert GL (1993). Invasive disease caused by multiple clones of *Corynebacterium diphtheriae*. Clin Infect Dis.

[30] Romney MG, Roscoe DL, Bernard K, Lai S, Efstratiou A, Clarke AM (2006). Emergence of an invasive clone of nontoxigenic *Corynebacterium diphtheriae* in the urban poor population of Vancouver, Canada. J Clin Microbiol.

[31] Ott L, Holler M, Rheinlaender J, Schaffer TE, Hensel M, Burkovski A (2010). Strain-specific differences in pili formation and the interaction of *Corynebacterium diphtheriae* with host cells. BMC Microbiol.

[32] Mandlik A, Swierczynski A, Das A, Ton-That H (2007). *Corynebacterium diphtheriae* employs specific minor pilins to target human pharyngeal epithelial cells. Mol Microbiol.

[33] Santos LS, Sant'anna LO, Ramos JN, Ladeira EM, Stavracakis-Peixoto R, Borges LL, Santos CS, Napoleao F, Camello TC, Pereira GA (2015). Diphtheria outbreak in Maranhao, Brazil: microbiological, clinical and epidemiological aspects. Epidemiol Infect.

[34] Kneen R, Pham NG, Solomon T, Tran TM, Nguyen TT, Tran BL, Wain J, Day NP, Tran TH, Parry CM (1998). Penicillin vs. erythromycin in the treatment of diphtheria. Clin Infect Dis.

[35] Mina NV, Burdz T, Wiebe D, Rai JS, Rahim T, Shing F, Hoang L, Bernard K (2011). Canada's first case of a multidrug-resistant *Corynebacterium diphtheriae* strain, isolated from a skin abscess. J Clin Microbiol.

[36] FitzGerald RP, Rosser AJ, Perera DN (2015). Non-toxigenic penicillin-resistant cutaneous *C. diphtheriae* infection: a case report and review of the literature. J Infect Public Health.

[37] Sheldon JR, Heinrichs DE (2015). Recent developments in understanding the iron acquisition strategies of gram positive pathogens. FEMS Microbiol Rev.

